# Factors associated with delayed diagnosis of pulmonary tuberculosis in Chitwan district of Nepal

**DOI:** 10.1371/journal.pgph.0005718

**Published:** 2026-01-08

**Authors:** Susmita Sharma, Jiwan Kumar Poudyal, Varun Kumar Sharma, Sumitra Parajuli, Govinda Prasad Dhungana

**Affiliations:** 1 Shree College of Technology, Department of Public Health, Chitwan, Nepal; 2 Department of Public Health, Noida International University, Greater Noida, Uttar Pradesh, India; 3 Department of Biotechnology and Microbiology, School of Sciences, Noida International University, Greater Noida, Uttar Pradesh, India; 4 Bharatpur Hospital, Nursing College, Chitwan, Nepal; 5 Department of Statistics, Birendra Multiple Campus, Trubhuvan University, Kirtipur, Nepal; University of Sydney, AUSTRALIA

## Abstract

Pulmonary Tuberculosis (PTB) remains a major public health issue in Nepal and is among the top ten causes of death from a single infectious agent globally. Diagnostic delay refers to the time lag between the onset of symptoms and the confirmation of a correct diagnosis. Delayed diagnosis increases disease severity, prolongs infectivity, and hinders timely treatment. This study aimed to identify factors contributing to diagnostic delays of PTB in Chitwan district, Nepal. A cross-sectional study was conducted among 317 PTB patients receiving Directly-Observed Therapy short-course (DOTS) treatment across all DOTS centers in Chitwan district, using complete enumerative sampling. Data were collected using a semi-structured questionnaire through face-to-face interviews, then analyzed using SPSS version 22 with descriptive and multivariate analysis at a 95% confidence level. Of the 317 PTB patients, 42.6% experienced patient delay, 33.8% health system delay, and 58% total delay. The median delays were 25 days (patient), 5 days (health system), and 30 days (total). Self-medication significantly increased the likelihood of patient delay (AOR = 5.893, 95% CI: 2.133–16.285), as did lack of TB knowledge (AOR = 3.355, 95% CI: 1.603–7.018), poor economic status (AOR = 2.149, 95% CI: 1.109–4.162), and domestic preoccupation (AOR = 2.017, 95% CI: 1.154–3.528). Health system delay was strongly associated with a lack of trained health workers (AOR = 66.202, 95% CI: 27.070–161.906), poor quality services (AOR = 1.102, 95% CI: 1.102–11.078), and distant health facilities (AOR = 4.830, 95% CI: 1.554–15.017). The study identified significant diagnostic delays in Pulmonary Tuberculosis, primarily influenced by self-medication, poor TB knowledge, low socioeconomic status, lack of trained health workers and domestic responsibilities. The findings emphasize the need for community awareness, socioeconomic support, and strengthened primary health services to promote early diagnosis and timely treatment for effective TB control.

## Introduction

Tuberculosis (TB), caused by Mycobacterium Tuberculosis, primarily affects the lungs, leads to pulmonary tuberculosis (PTB). Although TB is both preventable and curable, it has once again emerged as the leading cause of death globally attributable to a single infectious agent [1]. It persists as a significant global public health and one of the most fatal infectious diseases [2–7].

According to the Global Tuberculosis Report 2025, sustained progress in TB prevention, diagnosis and treatment has contributed to an overall improvement in the global TB situation. Nevertheless, an estimated 10.7 million new TB cases and 1.23 million deaths were recorded in 2024, corresponding to 11.5% case fatality rate [8]. This reflects a slight decline in incidence compared with 2023, when 10.8 million cases were reported in 2023 [9].

Recent data indicate that more than 80% of TB patients and fatalities occur in low and middle-income countries, with nearly 50% new cases in the South-East Asian region [10]. Despite this, United Nations aims for a World free of TB by 2030 [11]. Furthermore, according to the Global TB Report 2025, by 2035 the absolute number of TB deaths is projected to decrease by 95%**,** and the TB incidence rate is expected to decline by 90% [8].

Early diagnosis and appropriate treatment are critical aspects of epidemiological management of TB [2,4,6,12–14]. A number of studies have indicated significant delays in TB diagnosis and treatment due to factors associated with patient and health care settings [2,6,7,12,13,15,16]. TB patients propagate infection within the community, exacerbate disease severity, and correlate with an elevated risk of mortality [3,5,12,15,17,18]. It is estimated that of Pulmonary Tuberculosis (PTB) has the potential to infect 10–15 other individuals within a year and can remain infectious for 2–3 years of left untreated [15] and such delays are commonly reported in low and middle-income countries [19]. Even though a significant efforts to all forms of TB prevention and control, it persists as a top ten public health issue and leading causes of death in Nepal [20,21]. According to the National Tuberculosis Programme (NTP) Annual Report 2023, an estimated 117 TB patients occur per 100,000 population annually, yet the diagnosis and notification of only around two-thirds of these estimated cases indicate a significant gap in case detection [9,22]. A key obstacle to achieving elimination goals is the phenomenon of diagnostic delay, categorized into patient delay (symptom onset to first healthcare consultation) and health system delay (first consultation to confirmed diagnosis), the sum of which constitutes the total delay [23]. Delays in diagnosis and treatment initiation have severe public health consequences. These delays directly contribute to continued TB transmission, accelerated disease progression, and increased mortality [23,24]. Specifically, individuals with undiagnosed, smear-positive pulmonary tuberculosis (PTB) act as reservoirs, infecting an estimated 10–15 contacts per year, which perpetuates the epidemic [25]. Furthermore, this prolonged symptomatic period increases the economic burden on families through repeated healthcare visits and loss of income [26]. Despite national efforts such as active case finding and contact tracing, diagnostic and treatment delays persist, driven by a multitude of factors often identified in South Asian settings, including socio-demographic elements (low literacy, poverty, rural residence), patient behaviors (initial self-medication, consultation with informal providers, lack of TB knowledge), and systemic issues (limited access to diagnostic facilities, poor quality of care, and diagnostic errors [27,28].

In Nepal, the context is shaped by a diverse topography, a mix of public and private healthcare providers, and socio-cultural beliefs that often influence health-seeking behavior. Although the National TB Control Program (NTP) has emphasized the Directly Observed Treatment Short-course (DOTS) strategy, primarily relying on passive case finding, it has successfully expanded access to free diagnosis and treatment through its network of DOTS centers. Despite these advancements, Nepal has not yet met the End TB targets [20]. Consequently, despite systematic program implementation and strategic initiatives, diagnostic delays persist as a significant programmatic challenge, with the specific drivers of these delays varying significantly across regions due to underlying cultural, economic, and structural differences [28].

Chitwan district, with its unique profile as a hub of internal migration, a mix of urban and semi-urban populations, and the presence of both advanced tertiary care hospitals and remote primary health care outlets, presents a critical and representative setting for such an investigation. The convergence of diverse populations in Chitwan likely creates a complex dynamic for TB care seeking and provision. Despite the presence of major healthcare institutions locally, preliminary data suggests patients experience substantial delays in obtaining a pulmonary tuberculosis (PTB) diagnosis [29]. This indicates persistent barriers exist at both the individual and health system levels. Therefore, this study aims to determine the patient, health system, and total delays in the diagnosis of pulmonary TB and to identify the key factors associated with these delays in the Chitwan district of Nepal.

## Methods

### Ethics statement

This study obtained ethical approval from Shree Medical and Technical College Institutional Review Committee (Ref#20230226-67). Permission was obtained from all municipalities and rural municipality of Chitwan District before data collection. Before data collection, objectives and all information were thoroughly briefed to each patient. Informed written consent was obtained from each patient and parental consent was obtained for patients under 18 years of age. Confidentiality, anonymity, and privacy of the information were maintained throughout the study.

### Study design and setting

A quantitative cross-sectional study was carried out in all DOTS centers of Chitwan District, Nepal. The research took place in Chitwan District, located in Bagmati Province in the southwestern region of Nepal. It has diverse geographical landscapes, ranging from hills to plains, and varied ethnic, cultural, socio-economic backgrounds, as well as urban, semi-urban and rural settings [30].

Tuberculosis (TB) ranks as the sixth leading cause of death in Nepal and shows a higher prevalence and fatality rate in the Chitwan district [31]. Comprehensive TB services are available throughout the Chitwan district under the National Tuberculosis Program. At the time of this study, there were 75 DOTS centers in Chitwan district. The district public health office coordinates these centers, which provides sputum microscopy, GeneXpert, chest x-ray and treatment at different health facilities. Treatment is offered free of cost through multiple DOTS centers and multi drug resistant cases are referred to specialized centers for advanced care [29,32,33].

### Study population

This study was conducted among all pulmonary tuberculosis (PTB) patients aged 15 years and above who were receiving anti-TB treatment and were registered under the National Tuberculosis Program (NTP) in Chitwan District, Nepal, between March and July 2023. The study included patients with pulmonary tuberculosis (PTB) who were diagnosed either microscopically or clinically, were already enrolled in anti-TB treatment, were able to communicate and had provided informed consent to participate in the study.

### Sample size and sampling technique

The sample population consisted of individuals who had a confirmed diagnosis of pulmonary tuberculosis and had initiated treatment in accordance with the national guidelines. Of the 347 pulmonary tuberculosis (PTB) patients included in the study population, 317 provided written informed consent. Therefore, the final sample size for this study was 317. A complete enumerative sampling technique was employed to select the sample from DOTS centers across Chitwan District.

### Tools and measures

A semi-structured questionnaire was developed by the researcher as the study instrument. To ensure its validity and clarity, a pre-test was conducted among 32 (10%) patients in Gaidakot Municipality, Nawalpur using Nepali language. As Nawalpur and Chitwan are adjoining districts with similar socio-demographic and health system characteristics, the pre-test was conducted in Nawalpur since all PTB patients in Chitwan were included in the main study.

Based on the findings of the pre-test and feedback from subject experts, necessary modifications were made to refine and finalize the questionnaire.

- Section A: Socio-demographic Characteristics: This section comprised 14 items to gather information on the demographic profile of the patients.
- Section B: Knowledge Assessment: This section included 11 questions designed to identify patients’ knowledge about the TB, treatment place, family history, choice of health care service.
- Section C: Factors Related to Delays: This section consisted of 12 questions aimed at identifying and assessing factors contributing to delays.

### Data collection, management and analysis

A quantitative data were collected through a structured questionnaire using face-to-face interviews among patients who were receiving anti-tuberculosis treatment regimens according to the national protocol [34]. Additional information on patients was obtained from a review of paper-based TB diagnosis, treatment, and referral registers provided by the Government of Nepal. The collected data were checked for completeness and accuracy, manually coded and organized prior to entry. The statistical Package for the Social Sciences (SPSS) version 22 was used for data entry and analysis. Descriptive statistics (frequency, mean and standard deviation) were calculated. Bivariate analysis was performed using the Pearson Chi-square test, and multivariate analysis was conducted using binary logistic regression at a 95% confidence level to identify factors associated with delay.

### Model validation

The performance of the logistic regression model was assessed using standard diagnostic tests. The Nagelkerke R^2^ statistics indicated the proportion of variation explained by the model. The Nagelkerke R Square values of 0.150 and 0.575 indicate that the predictors accounted for 15.0% and 57.5% of the explained variance in patient delay and health system delay, respectively. Likewise, the finding revealed that the Hosmer and Lemeshow Test (χ^2^ = 4.598, p-value = 0.800) was for patient delay and (χ^2^ = 5.905, p-value = 0.206) was for health system delay, which indicates the model was fit further analysis. According to the classification table, 40.7% of cases were predicted as patient delayed, with an overall accuracy of 67.5%. Similarly, in the health system delay model, 72.0% of cases were predicted as delayed, with an overall accuracy of 86.1%. Therefore, the model was considered appropriate and reliable for predicting patient and health system delay.

### Operational definitions

**Tuberculosis patient:** In this study, tuberculosis patient is any person who have met the diagnostic criteria (clinically or bacteriologically conformed) and has been initiated on anti-tuberculosis treatment from DOTS centers and registered in government treatment protocol. Bacteriological confirmed means-the positive smear microscopy or Xpert or culture for Macrobacterium Tuberculosis and clinically diagnosed means- no bacteriologic conformation but diagnosed by a qualified clinician based on symptoms, imaging, histology or supportive tests and started on full anti TB regimen [35].

**Patient delay:** It is the period from the onset of the first symptom(s) of suspected PTB (usually cough, fever, weight loss, night sweats etc.) to the date when the patient first contacted with a formal health care provider or facility (Doctor, health post or hospital). If it is more than 30 days then considered a patient delay [36]. This duration is measured based on patients self-reported history.

**Health system delay:** This delay considered as from the date of the patient’s first contact with any health service facility or provider to the date of the final diagnosis. If this period is > 7 days, it is considered as a health system delay [37]. This duration is determined by reviewing health facility records, specifically from the patient’s first contact date at the health facility to the date of final diagnosis.

**Total delay:** In this study, it is considered as an individual who had entire time interval from the onset of TB symptoms to the initiation of anti-tuberculosis treatment. It is summing the duration of patient delay (>30 days) and health system delay (>7 days) [36,38].

## Results

### Socio-demographic characteristics of participants

Table 1 presents the distribution of the socio-demographic characteristics of the pulmonary tuberculosis patients. Thirty percent of the patients were aged 60 years or above, with a mean age of 47.07 ± 18.3 years. The majority were male (65.3%), belonged to the Janajati ethnic group (50.80%), lived in joint families (55.8%), and were married (71.9%). Nearly half of the patients had attained primary-level education (49.8%) and followed the Hindu religion (77.9%). Most patients (90%) resided in their own houses, of which half were classified as ‘sure’ and the remaining half as ‘raw.’ The majority used liquefied petroleum gas (LPG) for cooking, and slightly more than half (55.5%) were engaged in agricultural occupations,

**Table 1:** Socio-demographic characteristics of patients (n = 317).

Characteristics	Number (n)	Percent (%)
**Age (in years)**		
≤ 20	27	8.5
21-30	54	17.0
31-40	42	13.2
41-50	41	12.9
51-60	58	18.3
> 60	95	30.0
Mean ±SD (in years)	47.07 ± 18.3	
**Sex**		
Male	207	65.3
Female	110	34.7
**Ethnicity**		
Dalit	47	14.8
Janajati	161	50.8
Brahmin/Chhetri	87	27.4
Others	22	6.9
**Religion**		
Hindu	247	77.9
Buddhist	55	17.4
Others	15	4.7
**Family type**		
Single	140	44.2
Joint	177	55.8
**Marital status**		
Married	228	71.9
Unmarried	61	19.2
Widower	13	4.1
Widow	15	4.7
**Education level**		
Illiterate	55	17.4
Primary Education (Grade 1–8)	158	49.8
Secondary Education (Grade 9–12)	90	28.4
Higher education or above (Above grade 12)	14	4.4
**Occupation**		
Agriculture	176	55.5
Business	38	12.0
Private/Public job	28	8.8
Daily Wages	20	6.3
Others	55	17.4
**Home ownership**		
Own	284	89.6
On Rent	33	10.4
**Type of house**		
Sure House	159	50.2
Raw House	158	49.8
**Fuel used**		
Liquid Petroleum Gas	257	81
Firewood	60	18.9

- Sure house: a durable, well-constructed house built using brick and concrete with cement plastering.

- Raw house: a less durable house constructed using mud, bamboo and un-plastered materials.

## Univariate model results

### Types of delays and delay duration associated with TB diagnosis

**Fig 1:**
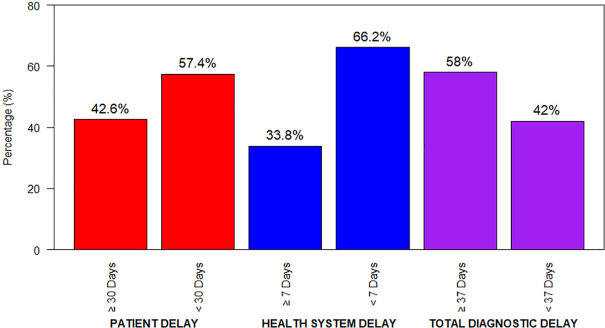
Patient distribution on the basis of different types of delays.

Different types of delays associated with the diagnosis of PTB were identified and presented in Fig 1. Among these delays, 42.6% were attributed to patient delay in seeking a diagnosis, while 33.8% were due to health system delays. Overall, 58% of patients experienced a total delay in their Tuberculosis diagnosis (Fig 1). Furthermore, the median patient delay, health system delay, and total delay were 25 days, 5 days, and 30 days, respectively.

## Multivariable model results

### a. Factors associated with patient delay

The findings of Table 2 revealed that among the predictors, only those with p-value ≤ 0.20 were considered in the multivariable logistic regression to control for the confounders. The results indicated that self-medication was six times more likely to result in patient related delay (AOR = 5.893, 95% CI: 2.133-16.285) compared to patients whose first choice of healthcare service was from a government health institution. Likewise, patients with no knowledge of TB were three times more likely to experience delay (AOR = 3.355, 95% CI: 1.603-7.018) compared to those with prior knowledge about TB. Patients with poor economic status were twice as likely to delay (AOR = 2.149, 95% CI: 1.109-4.162) compared to those with good economic status. Additionally, patients busy with domestic preoccupation were twice as likely to delay (AOR = 2.017, 95% CI: 1.154-3.528) compared to those free from domestic preoccupation. Likewise, marital status, family type, occupation, smoking habit, co-morbidity, family history, place of treatment, and stigma were insignificant predictors (Table 2).

**Table 2:** Factors which are associated with patient delay.

	Patient related delay (days)					
Characteristics	≥30 (n = 135)	<30 (n = 182)	UOR	AOR	P-value	95% CI
	n (%)	n (%)				
**Marital status**						
Unmarried	16 (26.2)	45 (73.8)	1	1		
Married	105 (46.1)	123 (53.9)	2.401	2.592	0.064	0.946-7.100
Widower/Widow	14(50.0)	14 (50.0)	2.812	2.364	0.210	0.615-9.088
**Types of family**						
Single	54 (38.6)	86 (61.4)	1	1		
Joint	81 (45.7)	96 (54.23)	1.344	1.366	0.276	0.780-2.395
**Occupation**						
Job	8 (28.6)	20 (71.4)	1	**1**		
Agriculture	78 (44.3)	98 (55.7)	1.990	**1.207**	0.721	0.431-3.379
Business	18 (47.4)	20 (52.6)	2.250	1.522	0.493	0.458-5.054
Wage-laborers	13 (65.0)	7 (35.0)	4.643	2.369	0.095	0.600-9.354
Others	18 (32.7)	37 (67.2)	1.216	2.491	0.141	0.738-8.412
**Smoking Habit**						
No	100 (40.3)	148 (59.7)	1	1		
Yes	35 (50.7)	34 (49.3)	1.524	1.417	0.286	0.747-2.686
**Choice of first healthcare service**						
Government Health Institutions	31 (31.0)	69 (69.0)	1	**1**		
Nearest pharmacies	35 (42.2)	48 (57.8)	1.623	1.467	0.262	0.793-2.910
By the Traditional Way	5 (55.6)	4 (44.4)	2.782	2.675	0.204	0.557-10.422
Private Health Facilities	43 (44.3)	54 (55.7)	1.772	1.872	**0.054**	0.980-3.365
Self-Medication	21 (75.0)	7 (25.0)	6.677	**5.893**	**<0.001**	**2.133-16.285**
**Co-morbidity**						
No	118 (40.8)	171 (59.2)	1	**1**		
Yes	17 (60.7)	11 (39.3)	2.240	1.853	0.182	0.749-4.584
**Family History of TB**						
No	127 (44.4)	159 (55.6)	1	**1**		
Yes	8 (25.8)	23 (74.2)	0.435	0.600	0.283	0.236-1.525
**Reasons of delay**						
Knowledge about tuberculosis						
Yes	130(71.4)	52(28.6)	1	1		
No	113(83.7)	22(16.3)	2.055	3.355	**<0.001**	1.603-7.018
Unaware of place of treatment						
No	44(24.2)	138(75.8)	1	1		
Yes	37(27.4)	98(72.6)	1.184	1.590	0.141	0.857-2.951
Economic status						
Good	33(18.1)	149(81.9)	1	1		
Poor	35(25.9)	100(74.1)	1.580	2.149	**0.023**	1.109-4.162
Domestic Preoccupation						
No	59(32.4)	123(67.6)	1	1		
Yes	58(43.0)	77(57.0)	1.570	2.017	**0.014**	1.154-3.528
Stigmatization						
No	9(4.9)	173(95.1)	1.353	0.566	0.422	0.141-2.271
Yes	5(3.7)	130(96.3)	1	1		

Nagelkerke R^2^ = 15.0% and Hosmer and Lemeshow Test (χ² = 4.598, p-value = 0.800).

### b. Factors associated with health system delay

The findings revealed that among the predictors, only those with p-value ≤ 0.20 were considered in the multivariable logistic regression to control for the confounders. The results indicated that a lack of trained health workers was sixty-six times more likely to cause health system delay (AOR = 66.202, 95% CI: 27.070-161.906). Similarly, patient who reported a lack of quality health services were significantly more likely to experience health system delay (AOR = 1.102, 95% CI: 1.102-11.078). Health facilities far from the residence were also more likely to cause of delay (AOR = 4.830, 95% CI: 1.554-15.017). Furthermore, the unavailability of health workers, lack of diagnostic facilities, and lack of effective supervision were insignificant predictors for health system delay (Table 3).

**Table 3:** Factors which are associated with health system related delay.

Factors of health system delay	Health System related Delay		UOR	AOR	p-value	95% CI
	Yes (≥7 days) n(%)	No (<7 days) n(%)				
Lack of trained health worker	77(72.0)	30(28.0)	42.350	**66.202**	**<0.001**	**27.070-161.906**
Lack of quality of health service	22(20.6)	85(79.4)	3.365	1.102	**0.034**	1.102-11.078
Health facilities far from residence	8(7.5)	99(92.5)	1.131	4.830	**0.006**	1.554-15.017
Not availability of health worker	1(0.9)	106(99.1)	0.981	4.172	0.293	0.291-59.801
Lack of diagnostic facilities	33(30.80)	74(69.20)	0.368	2.151	0.083	0.905-5.115
Lack of effective supervision	6(5.6)	101(94.4)	3.059	2.806	0.260	0.466-16.899

Nagelkerke R^2^ = 57.5% and Hosmer and Lemeshow Test (χ² = 5.905, p-value = 0.206).

## Discussion

The primary aim of this study was to identify the factors associated with delays in the diagnosis of Pulmonary Tuberculosis (PTB). The findings indicated that 42.6% of patients had a patient-related delay, 33.8% encountered health system delays, and 58.0% experienced a total diagnostic delay. Correspondingly, the median patient delay was 25 days, while the median health system delay and total delay were 5 days and 30 days, respectively. Similar findings were reported in other studies in West Bengal India [15], Northwest Ethiopia [39] and Nepal [13]. However, some of studies conducted in Nepal [40,41], Bangladesh [42] and systematic review of low and middle income countries [43] found some variation for diagnostic delays of Pulmonary Tuberculosis. This discrepancy may result from differences in the study settings, variation in health system efficiency, methodological approaches, and socio-cultural factors as health seeking behavior, awareness and accessibility of diagnostic services.

In terms of socio-demographic characteristics of the study population, highest proportion of patients were aged 60 years and above, with mean age of 47.07 ± 18.281 years. More than 65% of the patients were male and predominantly followed Hinduism. These findings were consistent with the studies of Bangladesh [44] and Nepal [13,45], although the majority of Bangladeshi patients followed Islam. In contrast, studies from West Bengal, India [15] and Uganda [12] found younger study population (16–30 years) with Christianity as the predominant religion.. In our study, most of patients were married, depended on agriculture but still 17.4% were illiterate. These findings align with mixed method study from Ethiopia [46], Uganda [12], but contrast with studies from Bangladesh [44] where 65% of patients had at least primary or secondary education, were unemployed or involved in household work, and the study of Northwest Ethiopia [39] found that, most of patients were female, orthodox Christians and 29.1% were illiterate. Such variations may be attributed to methodological variation and socio-cultural context.

Our study employed both bivariate and multivariate analysis to find out the relationship between factors associated with delays for diagnosing pulmonary tuberculosis (PTB). In bivariate analysis, marital status, choice of healthcare provider, comorbidity and family history of tuberculosis showed statistically significant associations with patient delay based on Pearson’s Chi-square test (p=≤0.05). furthermore, multivariable logistic regression analysis revealed that self-medication, was nearly six times more likely to result in delay compared to patients whose first point of contact was a government health facility. This finding was consistent with studies conducted in India [47] and Cambodia [48], which suggest that primary healthcare services are often more accessible and responsive, influencing patients’ healthcare-seeking behavior.

In this study lack of knowledge on PTB was three times more likely odds to experience delay compared to those who has knowledge about PTB, which was relevant with the study of Kerala India [47], Eastern Mediterian [49], Myanmar [50] and Ethiopia [51] as that poor knowledge and awareness leads to delay in seeking diagnosis and treatment. Another study of Malawi [52] contradicted our study result as contributing factors of patient delay were primary education and knowledge on more than three weeks of coughing is a sign of PTB in West Bengal, India [15]. It found the different socio-demographic characteristics (age, residence, marital status etc) were the major contributing factors for patient delay. These variations might be due to differences in study areas, methodologies and level of awareness in issue between the countries.

In our study poor economic status was twice as likely to delay compared to those with good economic status, which is similar finding with the Arsi Zone, Ethiopia [51] and Nepal [40] where higher odds of poor economic status contributes to delay diagnosis. A study conducted in Western Nepal [41] found there is no any significant role of economic status for delaying diagnosis. Similarly, busy with domestic preoccupation was twice as likely to delay compared to those free from domestic preoccupation for diagnosis of PTB. A study held in Beira city, Mozambique [7] found that, farming, visiting first a traditional healer, low TB knowledge and coexistence of a chronic disease were associated with increased patient delay. However a study of Myanmar [50] found less than a middle school education level, working in temporarily, comorbidity with diabetes, and poor awareness were significantly associated with diagnostic delays.

Similarly, results indicated that a lack of trained health workers at health facilities was the strongest predictor of health system delay (AOR = 66.202, 95% CI: 27.070-161.906), which is quite similarities with the study of Kerala, India [47] and Ethiopia [39] where the biggest contributing reasons to delay in diagnosis were the lack of health care-providers and patients who sought initial care from basic health institutions. However, distinct causes of health system delays were noted in the study of Ethiopia [51] and Nepal [40], where patients who visited two health workers and those who visited three or more and where farmer has the major reason for health system delay respectively. This might be variation in study methodology, sample size and study area. A lack of quality health services and health facilities far from the residence were significantly more likely delays in the health system. These findings are more likely to the study of India [17] where more than 1 kilometer of distance to reach health facility has higher probability to delay diagnosis.

Collectively, these factors contributing to delays in the diagnosis of pulmonary tuberculosis (PTB) may pose challenges to the implementation of National Tuberculosis Program and Strategic Plan to End Tuberculosis in Nepal [21,53]. Therefore, enhancing public awareness of PTB, improving healthcare accessibility and quality, training health workers and addressing socio-economic barriers are essential strategies to reduce diagnostic delay.

### Strength and limitations

The strength of this study lies in the use of validated tools and well-established methodology, developed after thorough consultation of relevant literature to ensure reliability. It also provides a comprehensive evaluation of the various factors contributing to delays in Pulmonary Tuberculosis diagnosis. Moreover, the study findings are consistent with national surveys, which enhances the generalizability of the results compared to previous studies.

Despite these strengths, it did not incorporate qualitative methods to explore patients’ perspectives in depth. Likewise, some information obtained from paper-based health records may have been missing or incompletely documented, potentially affecting data accuracy. Finally, information on the onset of symptoms was based on patient self-reporting. This reliance introduces the possibility of recall bias, which may influence the accuracy of factors associated with delays in tuberculosis diagnosis.

## Conclusion

The main aim of this study was to identify factors associated with delayed diagnosis of pulmonary tuberculosis. The findings revealed substantial delays in TB diagnosis, with approximately half of the patients experiencing a patient-related delay of 30 days or more and one-third encountering health system delays. Overall, a large proportion of patients experienced total delays, indicating a serious challenge in timely TB detection. Key determinants of patient delay included self-medication, lack of knowledge about TB, low socioeconomic status, and domestic responsibilities. These findings underscore gaps in both patient health-seeking behaviors and the healthcare delivery system. The study highlights the urgent need for targeted interventions to reduce diagnostic delays, including community awareness campaigns, socioeconomic support, and strengthening primary healthcare services through adequate staffing, training, and improved accessibility. Early diagnosis and timely initiation of treatment are essential to reduce TB transmission and achieve national and global TB control targets.

## Supporting information

S1 Data Data. (XLSX)
